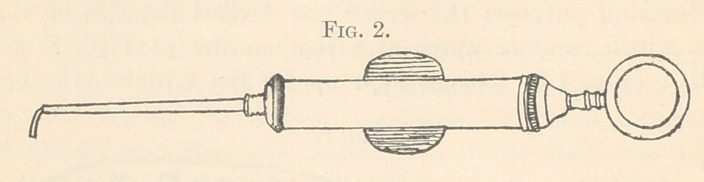# Oral Hygiene

**Published:** 1902-12

**Authors:** George F. Eames

**Affiliations:** Boston, Mass.


### ORAL HYGIENE.[1]

BY GEORGE F. EAMES, M.D., D.D.S., BOSTON, MASS.

To secure a healthy condition of the human mouth and maintain it in that condition involves much, not only in the variety of methods by which this may be accomplished, but in its beneficent results, not alone to the oral cavity itself, but to the entire organism.

These considerations may be classed under two heads,—viz., *methods* and *results*. Let us first consider " results;" that is, to put it in the form of a question, What results are desirable in order to secure the one grand achievement, a healthy mouth, the accomplishment of which underlies the whole practice of dentistry?

---

[1] Read before the Section on Stomatology, American Medical Association, Saratoga Springs, June 10, 1902.

As one of the first results, we may seek to obtain clean teeth, and by that we mean teeth that *are* clean,—clean not alone for the sake of their appearance, but with the object of improving their structure, of preventing decay, of invigorating adjacent tissues, and of securing the beneficial results which will come to other organs and to the entire system.

The idea has prevailed too generally that the mouth-cleansing done by the patient, that is, the cleansing of the exposed surfaces of the teeth and other accessible portions of the mouth, has no influence over the inaccessible places between the teeth. This the writer believes to be erroneous to a considerable extent, for the reason that, surrounding organs and tissues being in a state of health, contiguous parts must partake in some slight degree of the vitality and resistance which obtains in the healthy tissue. On the other hand, if those parts accessible to the patient are in a state of disease due to neglect, it follows that in some of the five ways in which disease is transmitted from one part of the body to another it will travel to those parts which can be reached only by the dentist, thus accelerating decay there, much to his discomfort.

The healthy mouth will not emit a foul odor. By this is not meant the temporary odoriferous breath which is occasioned by partaking of the "festive" onion, or other articles of food or drink.

What is known as a "bad breath" is caused by a putrefactive process going on somewhere. Our object should be to locate this and remove the condition if possible. Decay of the teeth, alveolar necrosis, destructive inflammation of the gums, malocclusion, and loss of teeth are at once conspicuously apparent to the eye and need no comment, but those horribly offensive masses that are often lodged in the crypts of the tonsils are frequently overlooked. And still more remote and difficult to observe are nasal polypi, ozæna, the foul secretions and lodgements which present in the pharyngeal tonsil, and other chronic inflammatory conditions of the nasal and post-nasal space. All these affect injuriously the oral cavity, by the mingling of secretions, by the transmission of bacteria, by the extension of the inflammatory process by means of continuity of tissue, and, more remotely, by the depreciation of the general health which must ensue.

The mouth is a swift and sure indicator of many general diseases, and especially of gastric derangements, which are manifested not only by the oral sensations of the patient and the appearance of the tongue, but by the condition of the saliva. The ropy and offensive condition of the saliva has often been noticed by the writer in connection with diabetes and gouty and rheumatic conditions, and he much appreciates the statement of Michaels that the saliva varies greatly in the facility with which putrefaction takes place in different specimens. Indeed, for the great advance in our knowledge of the saliva, of the way in which it is influenced by abnormal conditions, and of the use which may be made of it in the diagnosis of diseases very great praise is due to Dr. Michaels, of Paris.

It is also a cause for much satisfaction that the furtherance of this important work is in such able hands as those of Professor Edward C. Kirk, of Philadelphia.

That the oral secretions may become so vitiated as to constitute an important factor in producing decay of the teeth and disease of the structures which support and surround them will go undisputed, as will also the proposition that these secretions may be controlled to a great extent by systemic and local treatment, thus mitigating the evils just mentioned.

The tongue is often covered with infectious material, and when deeply fissured forms a surface not easily kept clean.

There are also many general diseases which contribute either wholly or in part to the above-named pathological conditions, or oral disease may be responsible for the general condition.

William B. Keyes, of London, has contributed valuable information on this subject. He enumerates several cases in which injurious effects were produced locally upon the stomach by means of infectious material from the mouth which was constantly being swallowed. He also cites cases illustrating the systemic effects resulting from the absorption of toxins. It seems appropriate here to call attention to the necessity for the education of the physician dentally and for the education of the dentist medically, and for consultation between the medical specialist in dentistry and the medical specialist in those conditions which have a pathological relation to the oral region.

It is not necessary to enlarge upon this subject before *this* section of the American Medical Association. You are all wise, therefore the word which I have spoken is sufficient. In fact, the writer wishes to avoid making a lengthy paper by repeating much that has been written on oral hygiene, but rather to give as far as possible the newer thoughts, relating especially to treatment.

Let us now consider the "methods" in the treatment of the various conditions which have been named in the foregoing portions of this paper.

The patient on rising should rinse the mouth several times with water, and follow this by brushing the teeth, using brushes, powder, and washes selected by the dentist. The teeth should be brushed for three minutes, or for a length of time suggested by the dentist as a result of his knowledge of the needs in each particular case. The patient should have several brushes, one containing rubber bristles. The cereal powders suggested by Dr. M. H. Fletcher seem to possess qualities which should commend them highly. The teeth should be brushed in all directions, including a rotary motion, with the object always of removing any foreign substances clinging to their surfaces.

The brush should be wet with an antiseptic wash, and plenty of powder should be used. It has been advocated that the brush be used dry, but of what advantage can this be? It will, after a little use in the mouth, become wet in some degree, and to use it in this pasty condition would be neither the wet nor the dry process. The dry brush with powder would, with extra power, remove particles from the exposed surfaces, and would produce a high polish, but parts not receiving this extra force would not be cleansed so well as by the wet process, provided enough powder is used and the bristles of the brush are sufficiently stiff.

The gums also should be brushed, but for this purpose, in the writer's opinion, a brush with rubber bristles is best, using it wet either with antiseptic wash or with vaseline, one ounce; menthol, three grains. If the condition of the mouth require it, the gums may also be rubbed with the tips of the fingers, on which the above preparation of vaseline has been placed. After this, especially at night, the following preparation may be pushed in between the teeth with the balls of the fingers.

℞ Vaseline, ℥i;

Cera alba, ℥ss;

Hydronaphtol, gr. xv;

Oil of cinnamon, gtt. ii.

Finally the mouth may be rinsed with some antiseptic solution.

Toothpicks may be used after meals, and may consist of wood, quill, or of metal. Gold toothpicks, properly shaped, are the best, in the writer's opinion. Careful instruction, however, should be given the patient as to the proper use of these agents, pointing out the danger of wounding the gums, of leaving particles of wooden picks between the teeth, and of using too much force with the metal ones.

Besides these the patient may use the stick and pumice, he being properly instructed in such use. This may not be indorsed by all practitioners, but the writer has often averred that the possibilities of what the patients may do for themselves under proper advice and instruction are very far from being reached. One patient in particular, who had been following my advice in this matter, said, on being reminded of a coming appointment, "There will be nothing to do. I'm a regular Fiji Islander," exhibiting the stick which he had been using. We had previously been talking of the custom of the South Sea Islanders, who use their spare time in rubbing their teeth with a stick, and of the benefit with which Americans might follow their example.

The writer has advocated in the past the cleansing of the tongue as a part of the necessary mouth toilet. He wishes now to modify his statements on that subject. He advises that the tongue be cleansed when necessary, by means of a brush with rubber bristles, and never with the stiff hair bristles which have been used for cleansing the teeth.

For diagnostic purposes, it would seem that any coating or deposit on the tongue should be left undisturbed; and when a diagnosis has been made, the scientific method of cleansing the tongue would be to correct the systemic and local conditions which caused the deposit. Meanwhile, if deemed necessary, the local cleansing of the tongue may be carefully done.

The disadvantages of scraping or scrubbing the tongue, so far as the writer has observed, are (1) the danger of getting bristles into it and causing inflammation and distress, and (2) the liability of infecting the tongue by the penetration of bristles loaded with bacteria in proportion as this organ needs cleansing. Small fissures or denuded surfaces are especially liable to become infected.

The tongue should be self-cleansing, and while it is not possible, perhaps, under the high state of so called civilization in which we are living, to bring about this desirable condition, yet much may be done in this direction by prescribing foods requiring prolonged mastication and use of the tongue; in fact, by restoring its function. It is the *use* which is made of an organ which largely determines its hygienic condition.

If one must have liquids with food, let him take it after swallowing the food; he will then be obliged to masticate in order to make it possible to swallow.

The tongue, the mucous membrance of the mouth, and the gums are extremely sensitive to general disturbances, and often show the effects of a remote pathological condition long before any other symptoms are manifest, and, indeed, sometimes these oral expressions of disease are the only ones which may be recognized. The dentist may now step in to do what the patient cannot do, and he has a serious and important task to perform, for upon it depends to a great extent the physical welfare of the patient.

The spraying of the mouth and teeth with antiseptic solutions by means of compressed air, the use of stick and pumice, the application of silver nitrate, the filling of cavities, the removal of deposits, the treatment of the gums, the correction of irregularities, the restoration of lost teeth, and the treatment of the various pathological conditions which may occur in the mouth are all familiar operations, and the writer would only urge that they be well and thoroughly done and leave it to the conscience of the operator.

Some of these operations and treatments, the writer thinks, may be improved; for instance, in cleansing and polishing between the teeth he has used instruments of his own design, with thin gold blades of various shapes, so very thin that they may be passed between or nearly between all teeth. These are dipped into some antiseptic solution, then into powdered pumice, and carried between the teeth, back and forth, first from the labial or buccal side, and then from the lingual, until the approximal surfaces are clean and smooth. These instruments can be carried where no stick can be used, and in the writer's practice this is a distinct advance over his former methods of treating these surfaces.

When the breath is impure, and the cause is not to be found in the teeth and surrounding parts, the tonsils should be inspected, and if pockets exist in the faucial tonsils they should be cauterized and the organ reduced in size if necessary. If adenoids are present the cause may exist there, and if so the redundancy of this tissue should be removed. The nose should also be examined, and polypi and catarrhal conditions should be treated. The cause of an impure breath may be very remote, and thorough examination of all the visceral organs should be made until the original cause is found. In these cases it is imperative that there should be a co-operation between the physician and the dentist.

In the treatment of inflamed conditions of the gum as well as for cleansing purposes the writer has devised douches of various forms which conduct water at a temperature of 110° F. to the mouth. (Fig. 1.) This is kept up for ten minutes, the patient

Fig. 1.

holding the head over a fountain cuspidor or bowl while the water runs out of the mouth. This idea was suggested to the writer while he was taking a shower-bath after a hot tennis match at Longwood. The water comes from the hand-hose with great force, and the stream was directed into the mouth, cleansing it most thoroughly and giving a delightful sense of comfort.

For the purpose of injecting a paste into pockets or between the teeth the writer has also devised an ointment syringe, the piston of which is driven by means of a screw. (Fig. 2.)

And now a word in relation to all that has been written regarding oral hygiene in the public schools. The dental examination of pupils is of undoubted value, but they seem to be regarded merely as material to be turned over to the dentist by the board of overseers.

However destitute parents may be, they have the rights of individuals, as citizens, as fathers and mothers, which should be respected, and their consent, not the pupil's, should be asked before a dentist, not of their choosing, shall insert instruments or fingers

Fig. 2.

into their children's mouths in the course of the examination of a hundred others. The father of small means has the right to ask that the same or equivalent antiseptic precautions be used for his children as for rich private patients, and his ignorance of these matters should be no reason for depriving him of the privileges belonging to any citizen.

Although many of the before-mentioned subjects have been treated briefly and perhaps inadequately, your essayist now comes to a stop, because "a wise man is like a spring lock, always more ready to shut than to open."

## Figures and Tables

**Fig. 1. f1:**
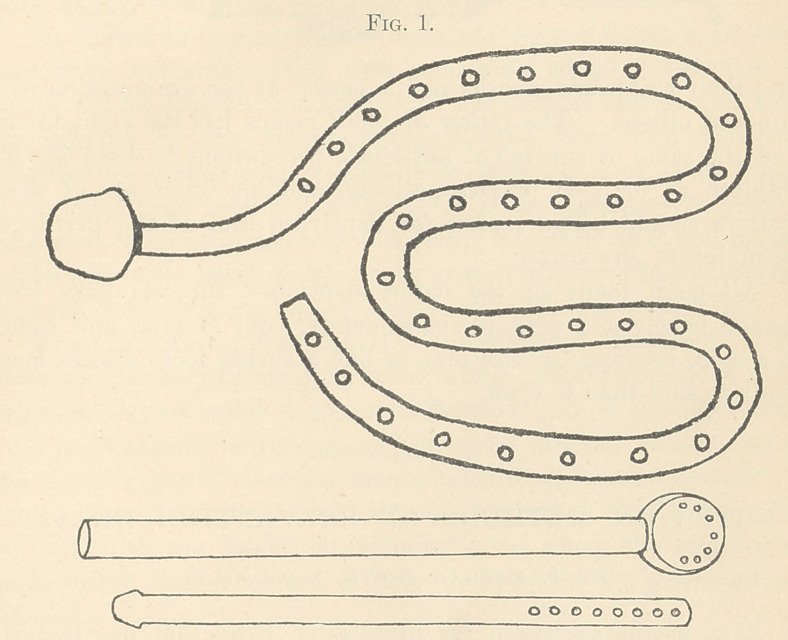


**Fig. 2. f2:**